# Text as signal. A tutorial with case studies focusing on social media (Twitter)

**DOI:** 10.3758/s13428-022-01917-1

**Published:** 2022-07-25

**Authors:** Eric Mayor, Lucas M. Bietti, Erick Jorge Canales-Rodríguez

**Affiliations:** 1 Department of Psychology, Division of Clinical Psychology and Epidemiology, University of Basel, Basel, Switzerland; 2 Department of Psychology, Norwegian University of Science and Technology, Trondheim, Norway; 3 Signal Processing Laboratory 5 (LTS5), École Polytechnique Fédérale de Lausanne (EPFL), Lausanne, Switzerland

**Keywords:** Signal processing, Signal analysis, FFT, FIR, Negative emotions, Positive emotions, Personal values, Presidential elections, COVID-19, Black Lives Matter

## Abstract

Sentiment analysis is the automated coding of emotions expressed in text. Sentiment analysis and other types of analyses focusing on the automatic coding of textual documents are increasingly popular in psychology and computer science. However, the potential of treating automatically coded text collected with regular sampling intervals as a signal is currently overlooked. We use the phrase "text as signal" to refer to the application of signal processing techniques to coded textual documents sampled with regularity. In order to illustrate the potential of treating text as signal, we introduce the reader to a variety of such techniques in a tutorial with two case studies in the realm of social media analysis. First, we apply finite response impulse filtering to emotion-coded tweets posted during the US Election Week of 2020 and discuss the visualization of the resulting variation in the filtered signal. We use changepoint detection to highlight the important changes in the emotional signals. Then we examine data interpolation, analysis of periodicity via the fast Fourier transform (FFT), and FFT filtering to personal value-coded tweets from November 2019 to October 2020 and link the variation in the filtered signal to some of the epoch-defining events occurring during this period. Finally, we use block bootstrapping to estimate the variability/uncertainty in the resulting filtered signals. After working through the tutorial, the readers will understand the basics of signal processing to analyze regularly sampled coded text.

**Supplementary Information:**

The online version contains supplementary material available at 10.3758/s13428-022-01917-1.

## Introduction

Language is the most successful communication tool, enabling the propagation and accumulation of human culture (Gelman & Roberts, 2017; Nowak & Krakauer, 1999; Pagel, 2009). Language in its multiple forms is more than a tool for communication (Boroditsky, 2019). It is also a window into the minds of members of communities (Pinker, 2007), from small groups (e.g., families) to large networks (e.g., nations). Associations between language use, perception, cognition, emotion, and behavior have been studied in psychology for decades (Barrett et al., 2007; Krauss & Chiu, 1998; Lupyan et al., 2020; Majid et al., 2004), including how such associations develop over time (Kopp, 1989).

Studies in psychology and computer science have shown that the linguistic style of social media users can be indicative of suicide (De Choudhury et al., 2016) and eating disorders (Walker et al., 2015), as well as measuring and predicting depression (Bathina et al., 2021; De Choudhury et al., 2013; Guntuku et al., 2017) and the side effects of antidepressant medication (Saha et al., 2021). Social media messages (e.g., Twitter) allow for larger sample sizes and a more inclusive data collection than self-reports. Further, social media research offers opportunities for reliable tracking of emotional and behavioral changes and their underlying emotional, cognitive, and social processes at population levels throughout space and time (Charlton et al., 2016; Ginsberg et al., 2009; Golder & Macy, 2011; Laranjo et al., 2015; Pang & Lee, 2008; Paul & Dredze, 2011; Wang et al., 2016).

Emotions are transient reactions to situations (Scherer & Meuleman, 2013), and their expression serves adaptive purposes, from the adjustment of the body to the immediate stimuli in the environment to the communication of survival information (Chapman et al., 2009; Rozin et al., 1994; Shariff & Tracy, 2011). Sentiment analysis refers to the coding of emotions expressed in text. It relies on categorical classification (e.g., positive, negative, neutral) or continuous rating of the extracted data (Puschmann & Powell, 2018). Sentiment analysis has been used to recognize emotions (Calvo & D’Mello, 2010; Cambria et al., 2017; Picard, 1997) and detect polarity (Cambria, 2016) in information from large unstructured datasets. Sentiment analysis has also shown that emotions expressed on social media feature 24-hour and 7-day patterns (Golder & Macy, 2011; Mayor & Bietti, 2021) and has allowed researchers to estimate the duration of positive and negative emotions as an effect of affect labeling, i.e., explicitly putting one’s feeling into words (Fan et al., 2019).

Periodicity is a property of many other psychological processes. For instance, cognition, especially memory, is impacted by circadian rhythms (Baddeley et al., 1970; Schmidt et al., 2007). Several stages are present in alternance during sleep (rapid eye movement and non-rapid eye movement phases, e.g., McCarley, 2007). During the day, people tend to daydream (mind wandering) with a roughly 90-minute periodicity (Othmer et al., 1969; Windt, 2021). Respiration, a systematic and periodic process, modulates neural transmission affecting cognitive processes (Varga & Heck, 2017). Increased cognitive load has been found to also affect respiratory patterns (Ebert et al., 2002; Grassmann et al., 2016). There also exist periodic patterns in the frequency in which people engage in interaction (Brown & Moskowitz, 1998; Gottman, 1979).

The detection of periodicity is a frequent interest in psychology, but most popular methods in text analysis do not permit the efficient estimation of periodicity, like circadian patterns (Golder & Macy, 2011). Indeed, associations with time are often not simply linear or even quadratic (Mayor & Bietti, 2021). There is thus a need for methods specialized in such investigations. Further, coded textual data usually feature significant noise, particularly if the underlying texts are only a few words long, such as those found in social media. To date, the coded data have primarily been used in their initial coded form in psychological studies or simply aggregated (e.g., averaged over periods of interest). Aggregation leads to a loss of information, and it averages measurement error. For instance, the influence of outliers might persist in the aggregated values. As such, aggregation is insufficient to attenuate noise in the data.

The main goal of this article is to introduce the reader to signal processing tools that can be used for the analysis of textual data extracted from social media. We do this in the form of a tutorial, including two case studies in the realm of social media analysis. The tutorial will enable psychology and computer science researchers to understand how signal analysis algorithms can benefit longitudinal text analysis and particularly social media analysis (e.g., removing noise in the data).

Treating text as signal entails the extension of signal processing tools, frequently employed in engineering, time-series analysis, and audio analysis to regularly sampled content-coded text. It affords several advantages overlooked by traditional methods, notably the following applications, which we cover in this manuscript as its main contribution: the denoising of time-series data, interpolating the data, detecting breakpoints, estimating periodicity, and obtaining uncertainty measures of such estimates through circular bootstrapping. Removing noise using signal analysis techniques is performed in most if not all studies using the electroencephalogram (e.g., Muthuswamy & Thakor, 1998) but has not yet been discussed in detail for regularly sampled textual data. The other basic methods in signal analysis mentioned above can be applied to coded texts, for instance, to detect periodicity patterns or visualize the data at different timeframes by removing measurement errors. Such applications have been proposed quite early in studying human behavior (e.g., Gottman, 1979) but were infrequently used when analyzing longitudinally collected texts (see the section titled Existing studies relying on text as signal in the Supplementary materials, S1).

Below we briefly mention popular methods in text analysis of interest for psychologists and computer scientists specializing in longitudinal text analysis. Readers might be interested in these techniques because the analysis of longitudinal datasets using the traditional methods in these fields often implies linearity in the associations of time with the variables of interest. Nevertheless, such associations are rarely linear, and the analyses using complex models (e.g., higher-order polynomials) may lead to overfitting (Yarkoni & Westfall, 2017) and lack of interpretability. The use of the approaches we detail below avoids these issues. Readers will learn about the outputs of the algorithms we will present and their parameters (i.e., arguments). We suppose the readers are active in psychology research and acquainted with Python programming (beginner level at least). The tutorial is still accessible to other readers who might need to familiarize themselves with Python through other sources^1^.

We then present two case studies (A and B) to demonstrate how such techniques operate in practice. In case study A, we examine emotional signal trajectories during the United States presidential election week of 2020, focusing on noise reduction using finite impulse response (FIR) filtering. In case study B, we analyze periodicity and patterns of changes in personal value signals during the turbulent period of November 2019 to October 2020 in the United States (COVID-19, Black Lives Matter) using fast Fourier transform (FFT) and FFT filtering, after relying upon data interpolation for the imputation of missing values. We finally discuss the limitations of the methods used in case studies.

### Popular methods of signal processing and analysis

This tutorial will use the following fundamental signal analysis and processing techniques: FIR filtering, interpolation, changepoint detection, FFT, FFT filtering, and bootstrapping. We have chosen these methods because of their widespread diffusion in signal processing applications, their relative simplicity, and the relevance of a tutorial for the treatment of coded text as signal (e.g., Birgham, 1988; Mohapatra & Mohapatra, 2017). We present these below and propose a description of other methods (S4) and include a glossary (S2) with complementary explanations of most of the terms we use here in the supplementary materials.

#### FIR filtering

In the context of one-dimensional discrete time series as input (e.g., a sampled signal), FIR filtering generates an output time series in which each value is computed based upon (1) itself and (2) preceding and (3) subsequent values in the input signal, depending upon a predefined sliding window length and filter type. A popular example is the moving average filter for which the weights along the window can be configured (e.g., triangular, flat, or another desired function). The advantage of triangular windows is to place a higher weight on the observation at the center of the window and linearly decrease the weights to observations further from it. On the other hand, all observations within the window are averaged without weighting when a flat window is used. FIR filtering distinguishes the underlying pattern in a time series from the random fluctuations by reducing the latter. Although it effectively filters the noise, a disadvantage of FIR filtering is a resulting shortening of the output time series (this is commented upon when discussing Fig. 2 in case study A) compared with the input time series, corresponding to the window length minus 1, because previous values do not exist for the first *wl* − 1 values in the signal.^2^

#### Interpolation

It allows filling in missing values in a sampled signal or resampling such signal to a higher temporal (or spatial) resolution. There are several interpolation methods, of which *linear* is a common type. Linear interpolation simply uses the existing values to the left and right of the values to be filled in and computes the latter linearly (e.g., the values 3 and 4 are respectively filled in the series 1, 2, NA, NA, 5, where NA represents missing values). *Polynomial* is another type of interpolation, where a given signal is interpolated by the polynomial that fits the data points in the signal.

#### Fast Fourier transform (FFT)

Fourier analysis originates in the idea that any analytical function can be approximated over a finite interval by taking a weighted sum of cosine and sine functions of harmonically increasing frequencies. Thus, the FFT allows us to decompose a discrete signal (of evenly spaced sampled points) from its original domain (usually time or space) to the frequency domain, as the sum of periodic sinusoidal waves with different frequencies. The FFT allows us to decompose a signal in its frequency components, like a prism that separates white light into different colors: one feeds in a signal, and it gives back the cosine and sine functions that, when added together, reconstruct the signal. This tool is employed in many fields, including vibration analysis, audio engineering, image processing, medical imaging, and time-series analysis. Two parameters are relevant for the FFT: (1) the sampling rate or sampling frequency fs of the measuring system, which is the number of samples collected in a given time (e.g., 1 second; 24 hours; 7 days), and (2) the total number of samples *N* (i.e., time points in the measured signal). From these two basic parameters (fs and *N*), other parameters of the measurement can be determined, including the Nyquist frequency fn (also called bandwidth), which indicates the theoretical maximum frequency that can be determined by the FFT (i.e., fn = fs/2). For example, if a signal is measured at a sampling rate of 100 Hz (i.e., 100 samples per second), then only frequency components up to 50 Hz can be theoretically estimated. This means that the sampling frequency must be at least double the highest frequency of interest in the signal (Holton, 2021). So, if one is interested in circadian (24-hour) changes in the signal, there should be at least two observations collected per day, at regular intervals. Conversely, the inverse FFT (IFFT; see case study B) allows us to reconstruct a signal from its frequency-domain representation (obtained after applying the FFT and performing potential filtering).

**Fig. 1:**
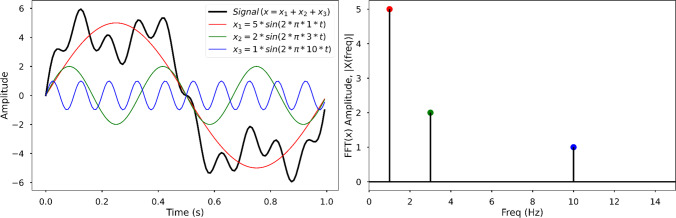
FFT example. The left panel shows a simulated composite signal (black line) that is the sum of three sine waves with amplitudes of 5 (red), 2 (green) and 1 (blue), and with frequencies of 1 Hz, 3 Hz, and 10 Hz, respectively (see the figure legend). The signal was generated using a sampling frequency of fs = 128 Hz, during a period of 1 s, for a total of *N* = 128 samples. The right panel depicts the FFT output of the composited signal. Only three components were identified, with frequencies and amplitudes matching those fixed in the composited signal. Each component was colored according to the corresponding sine wave

For instance, in Fig. 1, the signal represented by the thick black line is obtained by summing three sine wave functions with frequencies of 1 Hz (red line), 3 Hz (green line), and 10 Hz (blue line), and with amplitudes of 5, 2, and 1, respectively. The amplitude can be construed as the maximal distance from a point of equilibrium (indicated as 0 on Fig. 1). For example, an amplitude of 2 is twice as large as an amplitude of 1, as depicted in Fig. 1. Note that in this example, no noise was added to the data. For more details on FFT and additional examples, the reader is referred to chapter 24 in Kong et al. (2020) and Azad (2012, December 20). The output of the FFT is the amplitude of the signals with different frequencies, solely estimated from the original composited signal.

The oscillations of a pendulum provide an intuitive example of the amplitude and the frequency of a signal. In this example, the amplitude corresponds to the maximal distance from the point of equilibrium for each of its oscillations. Thus, the amplitude is highest when the pendulum is raised and released. It then decreases until the pendulum is steady. At that point, the amplitude is 0. The frequency of the oscillations of the pendulum can be defined as the number of times the pendulum crosses the point of equilibrium in a defined period.

In the realm of social media analysis, FFT analyses could, for instance, also be used to examine whether there exist patterns in posting frequency. One could imagine that social media users would post less during working hours, except for the weekends. If this were the case, the FFT analysis would show a stronger amplitude at the frequencies corresponding to one day and one week. Such an application complements the use we gave it in case study B as an illustrative example.

#### FFT filtering

Based on the inverse relationship between the FFT and IFFT, it is possible to attenuate or remove some of the frequency components in the signal. For instance, if after applying the FFT to the signal some of the resulting frequencies are removed, and the IFFT is applied, a filtered version of the original signal is obtained, which does not contain information about the discarded frequency component. This type of processing is commonly used to attenuate underlying noise in signals (by nullifying the amplitude of higher frequency components responsible for the fast signal changes in the time domain).

There are various FFT filters, including low-pass, high-pass, band-pass, and band-block. A low-pass filter blocks all frequency components above the predefined cutoff frequency (the amplitudes of frequencies above this cutoff are set to 0). An example application is the removal of movement artifact in electrocardiogram signals. In contrast, a high-pass filter removes all frequency components below the cutoff frequency. High pass filters are used in speakers for the removal of low frequency noise. A band-pass filter only allows frequencies to pass the filter within a chosen range, which is determined by the lower and upper cutoff frequencies. Bandpass filters are used in antennas as a mean to reduce the influence of different artifacts on the signal. There is another type of FFT filter called threshold filter. The signal components are not removed according to their frequencies but their amplitudes (i.e., those below a predefined threshold value). An important advantage of FFT filtering is that the resulting output signal is not shortened, as opposed to FIR filtering. For more details on FFT filtering, see Bevelacqua (2010, December 7). With regard to our example (see Fig. 1), an application of FFT filtering could attenuate noise due to the increased frequency of posting around the moment of global events. This occurs by applying a high pass filter.

#### Changepoint detection

This is concerned with identifying whether the behavior of the time series changes significantly and, if so, when. A change point indicates an abrupt change in the signal that may be related to a transition between two states in the underlying data generation process due to external events. This technique is helpful for modeling and interpreting time series in diverse disciplines, including climate change detection, speech analysis, and medical condition monitoring. For more details, see Truong et al. (2020) and the documentation of the *ruptures* Python toolbox (Truong, 2018, January 21) used in this tutorial.

#### Bootstrap

This is a resampling method that, given an initial signal, generates an arbitrary number of additional *pseudo* signals. It is based on mimicking the process of repeated sampling (with replacement) from a population by treating the sample as though it were the population. By computing the parameters of interest for each of the generated signals, it is possible to determine the empirical distribution of these parameters, from which confidence intervals and standard errors can be defined, allowing to characterize the uncertainty of the estimated parameters (Efron & Tibshirani, 1986).

A limitation of the classical bootstrap is that it performs poorly for time series (where each data point depends on previous data points) as it cannot replicate the correlation in the data. To incorporate this temporal dependency, other methods have been proposed, including the block-bootstrap, which is based on splitting the time series into consecutive non-overlapping blocks and using bootstrapping on each block. This resampling technique is mainly used when the data, or the errors in a model, are correlated. A crucial step in the analysis is the determination of the optimal block length, for which various automatic algorithms have been proposed; for further technical details, see Patton et al. (2009) and Politis and White (2004) and the documentation for the Python toolbox used in this tutorial (https://arch.readthedocs.io/en/latest/).

### Data requirements

The first requirement for coded text to be treated as a signal using FFT is that the measurement occasions should ideally be equally spaced (Cochran et al., 1967). For instance, once every hour, every day, more (or less) frequently, depending on the research questions. We note that rarely used algorithms exist for non-equally spaced measurement occasions (see Kircheis & Potts, 2019), resulting in additional complications. The nonuniform FFT is one of such algorithms used in medical imaging (see Liu & NGuyen, 1998). We also note that signal interpolation (see case study B) can resolve the issue of missing values in the signal. A second, related requirement is that there should be exactly one data point for each measurement occasion. Signal aggregation can resolve the issue of too much data. A recommendation is that the mean should be subtracted from each measurement value. The component frequencies are then less noisy and more easily computed. To compare the magnitude of change between dimensions, it is further recommended to standardize the signals using z-scores (after subtracting the mean from each measurement the resulting values are divided by the standard deviation). Last but not least, the number of observations should be at minimum two times larger than the highest frequency for which periodicity is presumed (Nyquist rate; see Sevgi, 2007).

## Tutorial and case studies

The case studies for this tutorial present the use and benefits of treating text as signal to remove noise from longitudinal coded textual data and examine patterns of change in such data. Here are the requirements for following the tutorial:

- Google Colaboratory (Colab) with
- Python 3 (included by default in Google Colab) for data analysis and
- Google Drive for hosting the data files.

For convenience, we use a Python 3 kernel in Google Colab to perform the case studies (see: https://colab.research.google.com). Python is a widely used programming language with probably the most comprehensive signal analysis capabilities that efficiently extend the content discussed in this tutorial to more advanced features. Code written in Python is generally understandable for people with experience with other languages (e.g., R). We used Google Colab notebooks in this tutorial to simplify things for users who do not have a Python integrated development interface (IDE) installed on their system, and because the use of notebooks has attested pedagogical value (e.g., Tan, 2021). Using Google Colab should be possible in less than 5 minutes, whereas the process can be much longer when installing an IDE locally and configuring it. We either import preinstalled modules or install them directly. Users who have such software installed can simply use it. To host the data, readers can use their existing Google Drive account or create a new one for the tutorial. The Open Science Framework repository for this project is: https://osf.io/dyfzv/. From there, readers can download the content of the folders Datasets and Colab notebooks and place all files in their Google Drive.

### Datasets used in the presented case studies

Datasets A and B are composed of coded tweets (aggregated values) collected in mid-November 2020. The tweets used to build the datasets for this study were collected and preprocessed as follows: We set our code to retrieve the timelines (up to 3,200 tweets) of 24,000 Twitter users located in one of the 160 most populated US counties. These users were selected using stratified sampling from a larger pool (Mayor & Bietti, 2021). The timelines of approximately 4000 users could not be retrieved (account deleted or changes in privacy setting). The tweets were preprocessed to remove emojis, special characters, links, and punctuation.

Dataset A covers a short period during which a major political event occurred in the United States. It comprises aggregated values of 317,861 emotion-coded tweets posted between November 1 and November 7, 2020. This dataset focuses on the week of the US presidential election of 2020. A total of 11,891 users from our pool tweeted during this period. Dataset B covers a considerably more extended period in which multiple major social, economic, and health events occurred globally (e.g., the COVID-19 pandemic). It comprises aggregated personal values (see below) from 7,917,884 coded tweets (from 18,317 users) posted between November 1, 2019, and October 31, 2020. Since December 2019, COVID-19 (including its health, social, and economic consequences) has become the most immediate global concern worldwide (Mirchandani, 2020) (for more details, see Supplementary materials, S3B).

The rationale for selecting these two datasets for the tutorial was the following. We wanted to include datasets associated with one local (2020 US presidential election) and one global (COVID-19 pandemic) epoch-defining event (Brown et al., 2009) for the selected population. Epoch-defining events are multi-layered and composed events, often unfolding over multiple time scales with macro-events composed of micro-events and specific actions organized sequentially. Thus, longitudinal analyses like the ones presented here are crucial for understanding the dynamics of such complex events.

Case study A addressed the time of the 2020 election week in the US when major political events occurred in a limited period and on a daily and hourly basis, The personal values coded tweets included in dataset B were posted during one of the most critical epoch-defining events in recent human history, i.e., the emergence of COVID-19 as a global pandemic. The propagation of the pandemic and subsequent measures taken by health authorities and political leaders, along with the accumulation of scientific evidence, imposed a different dynamic with events occurring on a daily, weekly, and monthly basis depending on their characteristics. This was the *zeitgeist* of the period when tweets were posted.

Tweets in dataset A were coded using the Linguistic Inquiry and Word Count 2015 (LIWC; Pennebaker et al., 2015). LIWC allows coding of more than 80 categories, among which we analyzed here the affective processes dimensions: positive emotion, negative emotion, anxiety, anger, and sadness. Choosing these categories allows us to exemplify our *text as signal* approach to identify variation in emotional dimensions in a limited period. The LIWC uses a simple word count approach, where words matching a lexicon (in which words and word stems are associated with preexisting categories) are coded for each processed document.

Tweets in dataset B were coded using the Personal Values Dictionary (Ponizovskiy et al., 2020). Personal values are believed to change relatively slowly (Milfont et al., 2016). The investigation of changes in personal values coded from tweets is unprecedented and choosing the Personal Values Dictionary also allows exemplify our approach in a more extended period. This dictionary is aimed at the investigation of human values expressed in text. Personal values can be divided into four quadrants: *self-enhancement* (composed of the values of achievement and power), *openness to change* (hedonism, stimulation, self-direction), *self-transcendence* (universalism, benevolence), and *conservation* (security, conformity, tradition) (Schwartz, 2010). Self-enhancement and openness to change have a personal focus, whereas conservation and self-transcendence have a social focus. Self-enhancement and conservation are anxiety-based values related to self-protection against a threat, and loss prevention, both are deemed to be grounded in extrinsic motivation. On the contrary, openness to change and self-transcendence are considered anxiety-free values related to self-expansion and growth, the promotion of gain, and grounded in intrinsic motivation.

In the next section, we employ case studies A and B to show the benefits of using signal analysis methods in the realm of social media analysis.

### Case study A: FIR filtering and emotional signals on Twitter during the week of the 2020 US presidential election

The learning objectives for case study A are (1) to set up a Google Colab notebook for the uses of the tutorial, (2) to perform FIR filtering on aggregated coding of textual data (for denoising the data), including the use of different settings of the filtering function (to introduce the reader to these settings), (3) to plot the resulting signals with different settings and in comparison with the unfiltered data, and (4) to examine automatically detected changes in the signal trajectories using changepoint detection. This allows the visualization of patterns in different filtered signals and illustrates the benefits of the methods. Additionally, changepoint detection will be used to present an automated analysis of abrupt changes in the signal.

For these purposes, we investigate the emotional signal trajectories during the US presidential election week of 2020 (see Supplement S3 for a description of the events). We link such variations to the specific events that occurred during the period. Readers can open the notebook *CaseStudyA.ipynb* from their Google Drive, and when prompted to select an application, choose Google Colab to open the file.

- *Step 1. Granting access to Google drive from Colab, installing and importing packages.* We first import the modules and functions necessary for this case study (i.e., *os, pandas, numpy*, *signal* from *scipy.signal, zscore* from *scipy.stats*, *plt* from *matplotlib.pyplot and drive* from *google.colab*). Then, we allow Google Colab to access Google Drive and load the dataset for case study A (Cell 1, last line):

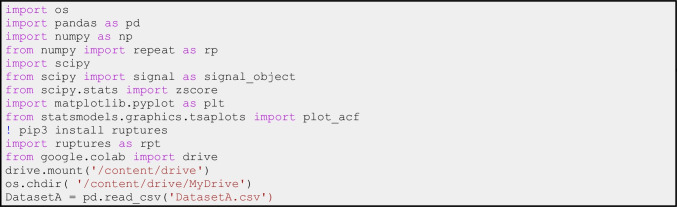

Running the cell can be done by clicking on the Play icon on its left or holding Shift and pressing Enter (Shift + Enter) while the cursor is in the cell. Running other cells can be done by repeating the Shift + Enter combination. Readers can run the cells as they read, or all at once.

After running Cell 1, a prompt appears: “Go to this URL in a browser: <URL>; Enter your authorization code: <input field>”. After the code from the URL is pasted in the required field on Google Colab, Google Colab has access to Google Drive and the dataset is loaded^3^. This procedure can be used for granting Google Drive access to Google Colab in any project requiring it.

- *Step 2. Centering and aggregating.* We center the relevant variables by user_id (in Cell 2). The resulting variables now represent change within users around their mean. We also aggregate these data by day, hour, and quarter-hour to obtain equally spaced observations with sufficient granularity. We will refer to these data as *emotional signals*. Finally, we create a date and time vector, which will be helpful later for plotting emotional variation through time. The centering procedure below can be used in other projects in which there are several observations per grouping variable (here the Twitter user).

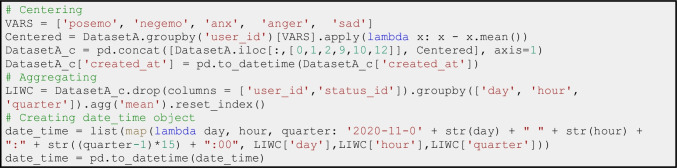

**Fig. 2:**
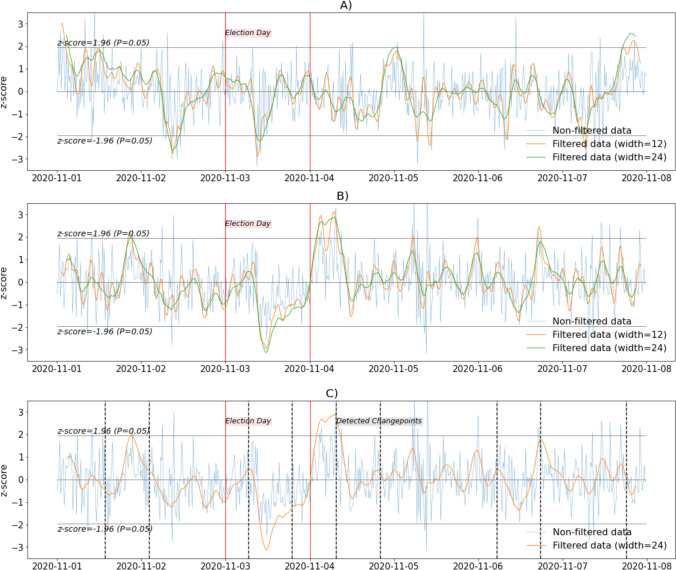
Variation in positive and negative emotions during the 2020 Presidential election week. **a** positive emotions, unfiltered data and FIR-filtered data, using triangular windows with window sizes of 12 and 24. **b** negative emotions, unfiltered data and FIR-filtered data, using triangular windows with window sizes of 12 and 24. **c** FIR-filtered data using triangular windows with a window size of 24 and detected changepoints. All signals were standardized and plotted as z-scores

- *Step 3. Function declaration.* We now define in Cell 3 our FIR filtering function, which we name *FIR()*. The function takes a signal, the type of the sliding window, and its length as arguments. This function can use several types of sliding windows. We also define a function for the filter parameters, *filter_parameters*(), and functions for displaying and formatting the figures: *PlotFormatter*() and *Plot_filtered_data*(). For the sake of brevity, we do not reproduce these functions here.
- *Step 4. Plotting the FIR-filtered emotional signals. Positive and negative emotions.* In Cell 4, we plot the emotional signals (in Panel A: positive emotions; and Panel B: negative emotions) filtered using window sizes of 12 and 24 (corresponding respectively to 3 and 6 hours, as each observation corresponds to 15 minutes). We chose these somewhat arbitrary values based on the length of the period of interest and because we were interested in changes that span several hours rather than short-lived changes. Denoising the data with such granularity, therefore, was deemed appropriate. We set the window type as "triangle" (meaning that values closest to the center of the window have a higher weight). Moreover, we apply a changepoint detection algorithm to the negative emotion signal (Panel C) to automatically identify shifts in the trajectory of this signal. All results are depicted in Fig. 2. We suggest that the reader pay close attention to the shortening of the filtered signals in the top and middle panels of Fig. 2a and b: The line corresponding to an FIR filter window width of 12 (orange) starts some pixels after the beginning of the unfiltered signal line (blue). The line for the FIR filter window of 24 (green) has an increased shortening. In both cases, the shortening corresponds to the window width. FIR filtering can be used in projects featuring continuously sampled observations.

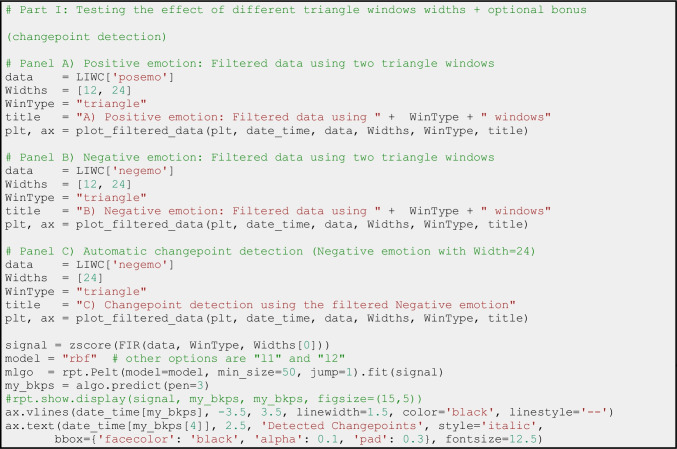



In Fig. 2a and b, we see that the unfiltered signals representing the variation in positive and negative emotions during the presidential election week are quite jittery and challenging to interpret. In contrast, we can better observe changes in positive and negative emotions from the FIR-filtered signals over the week. For example, a substantial dip on November 2, reaching two standard deviations below the mean for the considered period, followed by an increase on November 3 (Election Day), and other dips and increases are observed for the positive emotion. The lowest values occurred on November 2nd, 3rd, 5th, and 7th, which were statistically significant (i.e., smaller than the average, at *p* < 0.05) for the filtered data with a window size of 12. For the data filtered with a window size of 24, the significance was achieved only on November 2 and 3. The highest values occurred on November 1 and 7, as the presidential election outcome became more concrete.

There was also an interesting variation in negative emotions, with an increase by the end of November 1, a strong dip reaching more than three standard deviations below the average for the considered period on November 3 (Election Day, the lowest value observed), shortly thereafter followed by an increase reaching three standard deviations above this average on November 4 (the highest observed value). Note that these variations were statistically significant for both filtered signals. These patterns could not be seen using the unfiltered signal (see Fig. 2b). There was a difference of almost six standard deviations between the highest and lowest FIR-filtered values for positive and negative emotional signals.

Figure 2c shows the changepoints detected from the filtered negative emotions (dotted vertical black lines). The first occurred in the afternoon of November 1 and is related to an abrupt increase in the signal. The next changepoint occurred on November 2 and is associated with a substantial decrease in the signal. Notably, the third and fourth changepoints match the beginning and end of the voting period during Election Day.

**Fig. 3:**
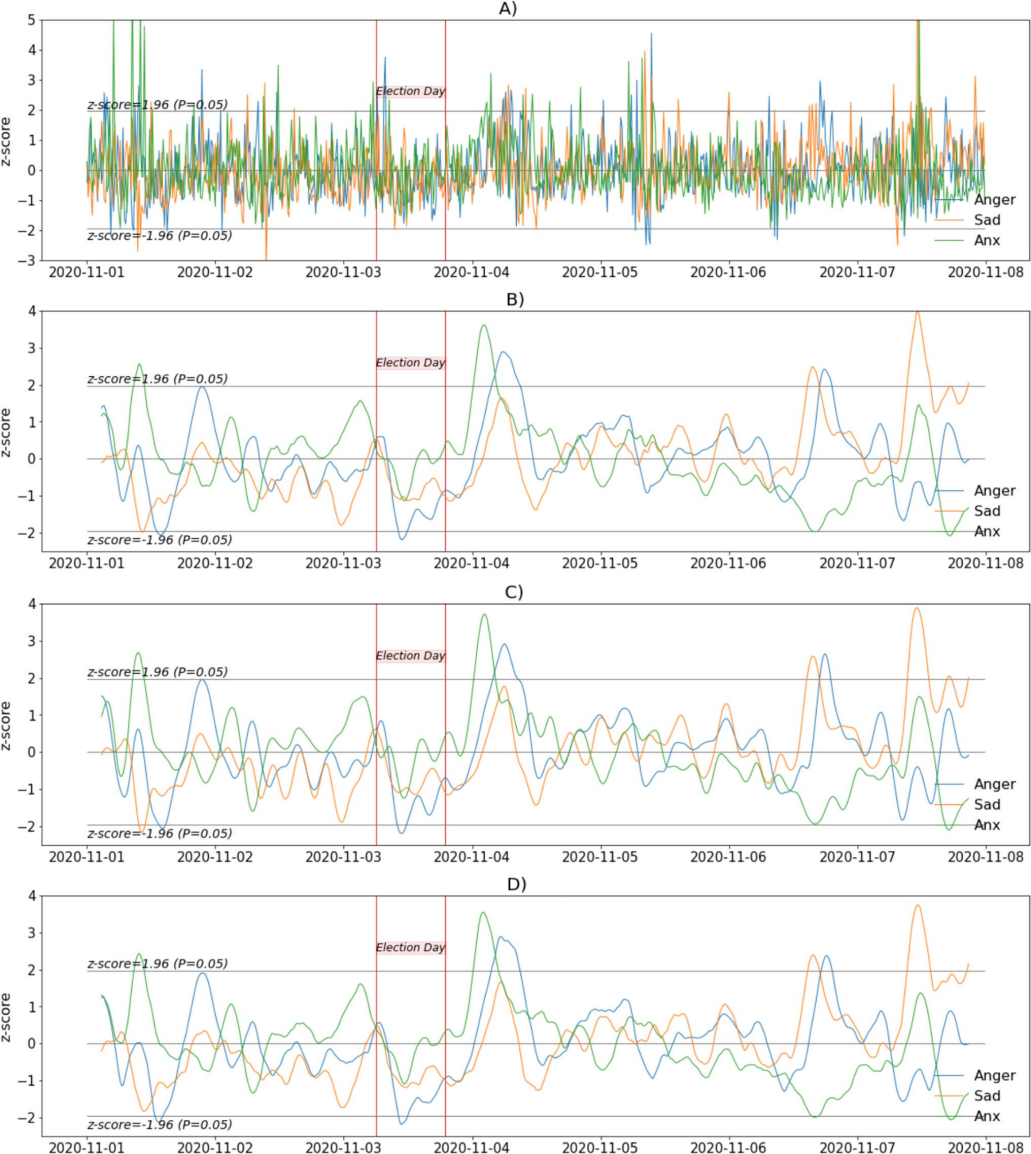
Variation in anger, sadness, and anxiety during the 2020 presidential election week. **a** Unfiltered data. **b** FIR-filtered data using triangular windows. **c** FIR-filtered data using Hann windows. **d** FIR-filtered data using cosine windows. All signals were standardized and plotted as z-scores. All filtered signals were filtered using the same window size of 24

- *Step 5. Plotting the FIR-filtered emotional signals. Anger, sadness and anxiety. *In step 5 (Cell 5), we plot the FIR-filtered signals for anger, sadness, and anxiety in Fig. 3. The unfiltered data appear in Fig. 3a. We used several windows for comparison (Fig. 3a: triangular windows; b: Hann window; c: cosine window). The values are standardized (z-scores) to obtain meaningful comparisons, and the same window size (i.e., 24) was used to filter the signals.

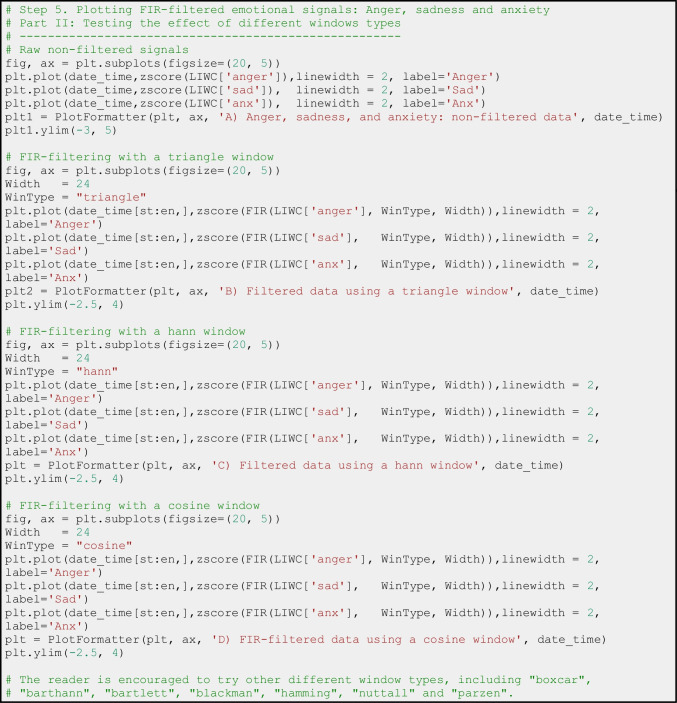



Changes in anger, sadness, and anxiety (FIR-filtered signals) can be observed in Fig. 3. In Fig. 3a, we provide the graph of the unfiltered signal, and in Fig. 3b−d, graphs for the triangular, Hann, and cosine windows, respectively. A notable peak in anger (three standard deviations above the average for the considered period) was observed on November 4. Anger was at its lowest on November 3 (Election Day; reaching two standard deviations below the average for the period) for all types of filters. Sadness varied, following a trend somewhat similar to anger until November 6th. On November 7, sadness increased to its maximum (three standard deviations above the mean for this period). Anxiety reached values around two standard deviations above the average for this period on November 1 and 3 and its peak value on the fourth (three standard deviations above the average for the period) and values around two standard deviations below this average on November 6 and 7 as the uncertainty regarding the outcome of the presidential election decreased. There were five standard deviations between the highest and lowest FIR-filtered values for these discrete emotional signals. Interestingly, although the three filtered signals (based on three window types) are not identical, they are very similar, and choosing any particular window type does not change the interpretation of the presented results.

#### Summary

After having been granted access to Google Drive from Colab and installing and importing the required packages for case study A, we explained how to load the dataset and the procedure that the reader should follow for centering variables and aggregating the data (i.e., emotional signals) to achieve equally spaced observations with enough granularity. Then, we defined the FIR filtering function, executed the FIR filtering of the emotional signals, and plotted the FIR-filtered emotional signals using different settings for window sizes and FIR filter types. We included a comparison of the results with and without the use of FIR so the readers could visualize the benefits of the method. We also used changepoint detection to automatically estimate shifts in the signal trajectories.

### Case study B: FFT and FFT filtering personal value signals in 12 months (November 2019–October 2020, before first COVID-19 wave to the beginning of the second wave)

In case study B, we will examine the use of interpolation (for the imputation of missing values), the FFT (for the analysis of periodicity in the signals), and FFT filtering (to denoise the data without signal shortening) to personal value-coded tweets from November 2019 to October 2020. This will allow us to examine the variation in the filtered signal in relation to epoch-defining events occurring during this period. We will also perform circular bootstrapping on the resulting signals (in order to estimate their uncertainty). The learning objectives of case study B are (1) to perform data imputation using signal interpolation within counties (so that each county is equally represented at all times), followed by aggregation over counties (so that there is one value per measurement occasion, a requirement explained in the introduction; (2) to examine the periodicity in personal value signals in these data (FFT; to determine which frequencies account for most variation in the signal); (3) to obtain data filtered from relatively high signal frequencies for which the explained variation goes beyond the interest of this analysis; and (4) to use circular bootstrap to obtain measures of the uncertainty of the resulting filtered signals. These steps will allow the reader to understand the application of FFT and related methods for the analysis of signals from repeatedly coded text. Notably, the reader will learn to determine at which frequency there is a more important change in the data, and how to filter out the impact of frequencies above those of interest.

We treated personal values coded from text as signal and examined their periodicity and how these evolved over 12 months. The original data for this case study are voluminous and would take a long time to process. Therefore, we already performed the aggregation by county, hour, and day. The unit of analysis is hence the hour, within-day and within-county. Due to the limited number of users for which the timelines were downloaded within each county, missing values were observed^4^.

Readers should first open the Colab notebook for this case study (CaseStudyB.ipynb).

- *Step 1. Granting access to Google drive from Colab, importing packages and loading the dataset.* As we did in case study A, before we load the dataset for case study B, we first need to allow Google Colab to access Google Drive (Cell 1, last line). We also want to proceed to the importation of the necessary modules and functions for the case study.

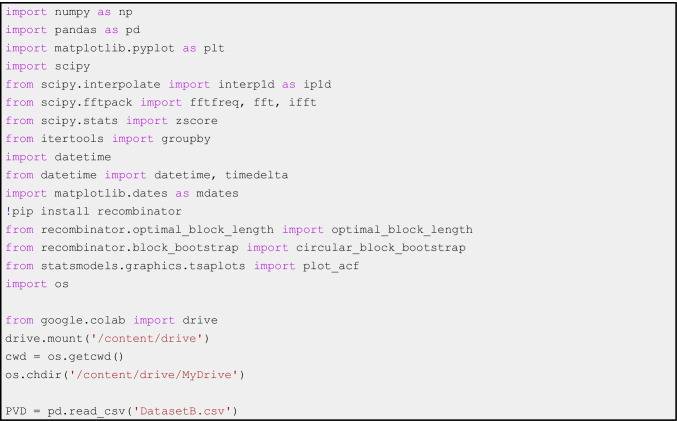

- *Step 2. Function declaration.* We declare a few functions in Cell 2, which, for the sake of brevity, are not reproduced here:
*to_wide()* creates a data frame containing the data of one variable, arranged by time (date and hour) in rows and county in columns.
*Interpolate()* will interpolate the data over one column using linear interpolation.
*Interp160()* will iteratively apply the function *Interpolate()* to the data of all counties, stored for each in one column.
*FFTgraph()* will plot the power (magnitude squared) of the frequencies of a signal.
*FFTfilter()* will perform low-pass FFT filtering of a signal (denoising) at the user-specified threshold.
*plotWithinTimeRange()* will plot observations within a user-specified time range for two variables.
*PlotFormatter1(), PlotFormatter2() and PlotFormatter3()* will be used to apply different formatting operations to graphs.
- *Step 3. Interpolate the data.* The functions *Interpolate()* and *Interpolate160()* we defined above require a wide dataset, where data for each county are contained in a different column in chronological order. Therefore, we first arrange the data in such form before interpolation using the *to_wide()* function. This function requires a vector indicating the measurement occasions on which to operate (named *Date_time* here). We performed all this in Cell 3 and checked that there were no missing values left in the data, which is the case.

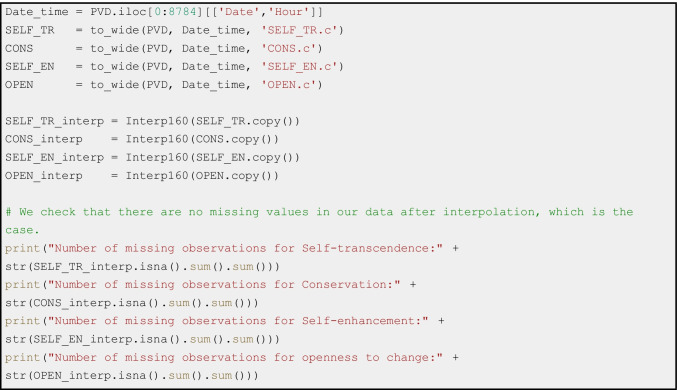

When using signal analysis, we recommend against using methods of imputation that do not consider the local, i.e., temporal, context of the data to be imputed, as it can be expected that such imputation methods will lead to a distortion of the signal. Further, these methods make assumptions that are challenging to meet for frequently sampled textual data (e.g., values missing at random). One example is that tweets are less regularly posted during the night, potentially leading to more missing values at the aggregate level at that moment. Beyond social media analysis, interpolating the data can be used in projects featuring data that have been regularly sampled but for which some observations are missing. We note that transforming a dataset from a long to a wide format is often a necessary step for longitudinal data analysis. The *to_wide()* function we provide in the notebook can be used for such purpose in other projects.

- *Step 4. Aggregation of over counties*. We now average the data over the counties (Cell 4) to obtain a single time series for each variable, representing each county equally.

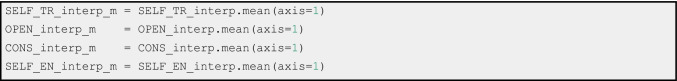

**Fig. 4:**
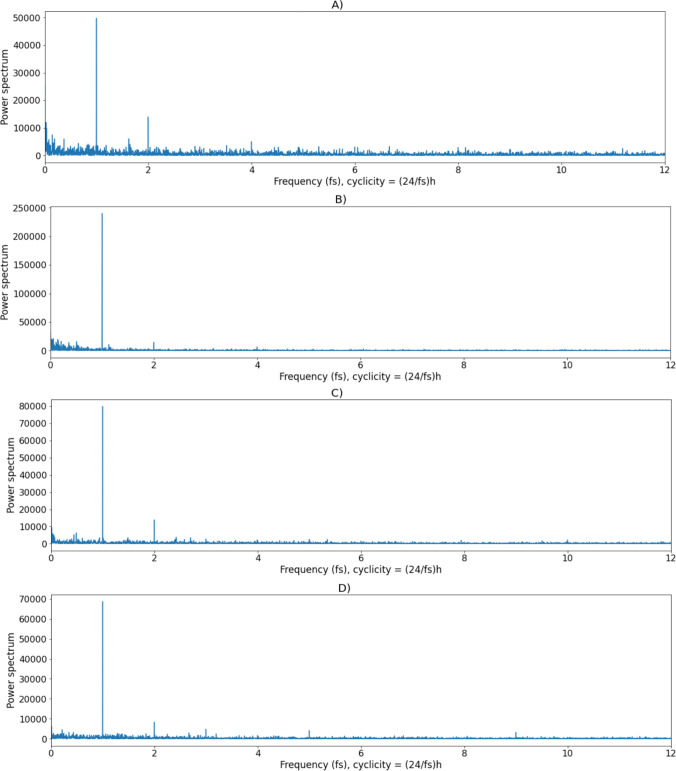
Spectral plots. **a** Self-transcendence. **b** Openness to change. **c** Conservation. **d** Self-enhancement

- *Step 5. Plotting the power of the frequencies in the signals (spectral plots).* Spectral plots provide information on the signal spectrum, i.e., which frequency has higher power. The signal is more strongly affected by frequencies with higher power. This hence provides information on the periodicity of the data. Let us examine the frequencies in each resulting variable using an FFT. In Cell 5, we produce the spectral plots of the personal value signals (see Fig. 4) at a sampling frequency of 24 (unit: day, one measurement occasion per hour). What is immediately striking in the four spectral plots is that the largest power (*y*-axis) is in all cases around 1 (*x*-axis), meaning that there is important circadian (24-hour, low frequency) periodicity in the signal of all personal values. The peak at 0 corresponds to a constant offset of the signal. One can also observe a smaller secondary peak at the *x*-axis value of 2, meaning that there is also a 12-hour periodicity in the signals. The values of the power spectrum for frequencies larger than 6, corresponding to a 4-hour periodicity, are small and so we can assume that there are no important cycles in the signals shorter than 4 hours. Note that the power spectrum is plotted in the range of frequencies up to 12, which is the highest (Nyquist) frequency that can be analyzed for this data, i.e., corresponding to periodic cycles of 2 hours or longer.

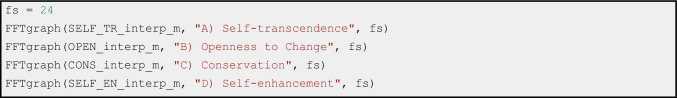



We note that we could have used other types of coded text treated as signals for our analysis using the FFT; an example is the affective dimensions of the LIWC (see case study A). We also could have set the unit of analysis to the week, in which case the sampling frequency fs would have been equal to 24 * 7. This would simply have changed the values on the *x*-axis.

- *Step 6. Removing circadian periodicity and plotting the self-transcendence signal*. The period of interest spans one year from November 1, 2019, to October 31, 2020. For example, we first examine the variation in the self-transcendence signal between November 15 and January 15, 2020, after removing circadian periodicity and higher frequencies, which we consider as noise because we are not particularly interested here in the changes happening at a faster time frame than a day. In other words, we remove circadian periodicity because variation related to more extended periods is blurred by substantial circadian variation. For this, we employ an FFT low-pass filter with a threshold of 0.5 (Cell 6). Remember that 1 represents circadian (24-hour) change, so 0.5 represents 48 hours (half the frequency of 1). We then plot the resulting filtered signal and the unfiltered signal for comparison. Such low-pass filtering could be applied to coded textual data at the participant level, in order to remove unwanted noise prior to performing other analyses, as routinely performed in EEG studies for instance.

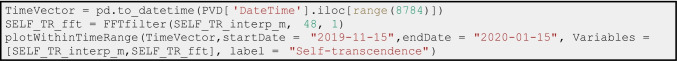

**Fig. 5:**
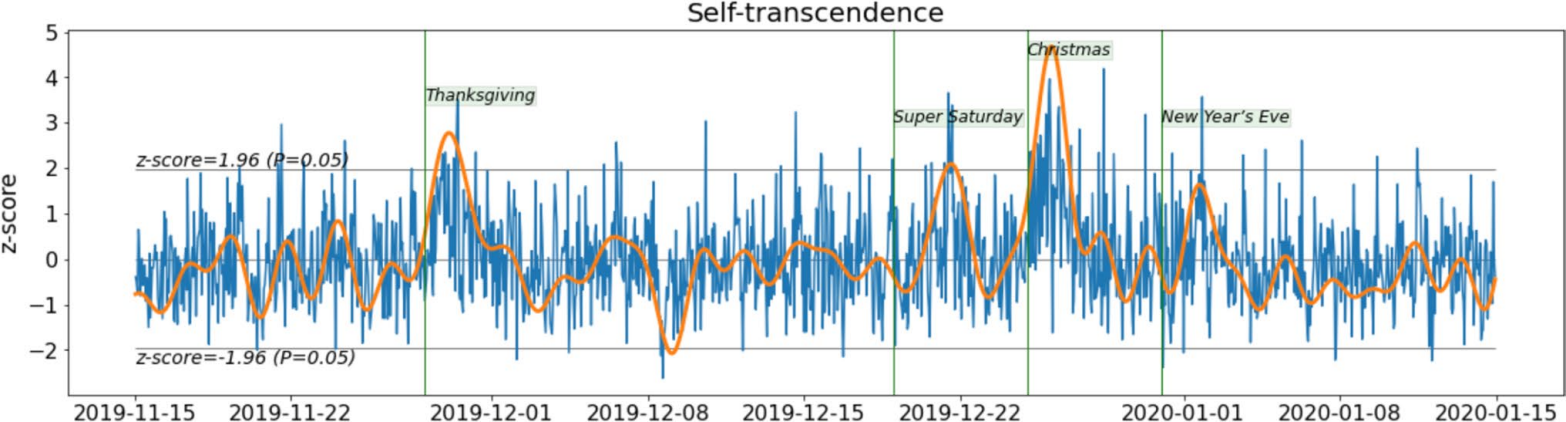
Self-transcendence between November 15, 2019, and January 15, 2020. Blue line: aggregated data after interpolation. Orange line: FFT-filtered data with periodicity beyond 48 hours removed

Loo﻿king at the orange line in the resulting plot (FFT-filtered data, Fig. 5), one can observe an increase in self-transcendence around Thanksgiving, Super Saturday (December 19, 2019), Christmas, and New Year’s Eve. It can be seen that the blue line showing the aggregated signal after interpolation is quite noisy in comparison, notably because of the influence of circadian patterns in the data (see the higher power of a frequency of 1 in the spectral plots, corresponding to one day, as mentioned above).

- *Step 7. Change in the personal values signals over 12 months: removing periodicity above one week.* In this example, we are not interested in changes that occur at time scales shorter than a week. Therefore, we apply an FFT low-pass filter that will remove the periodicity of the data above one week, i.e., a cutoff frequency of 1/7. We then plot the resulting signals two by two, first for conservation (blue) and self-transcendence (orange) in Fig. 5a, then for self-enhancement (blue) and openness to change (orange) in Fig. 5b. We include the indication of special dates (green and red vertical dotted lines).

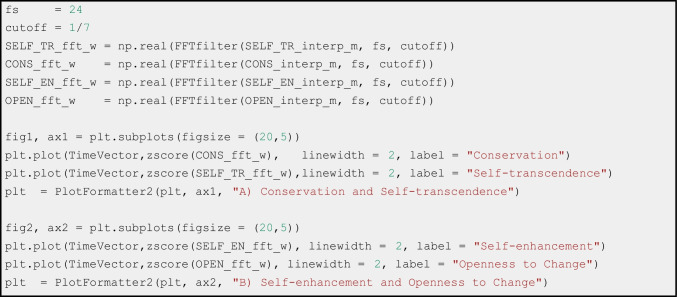

**Fig. 6:**
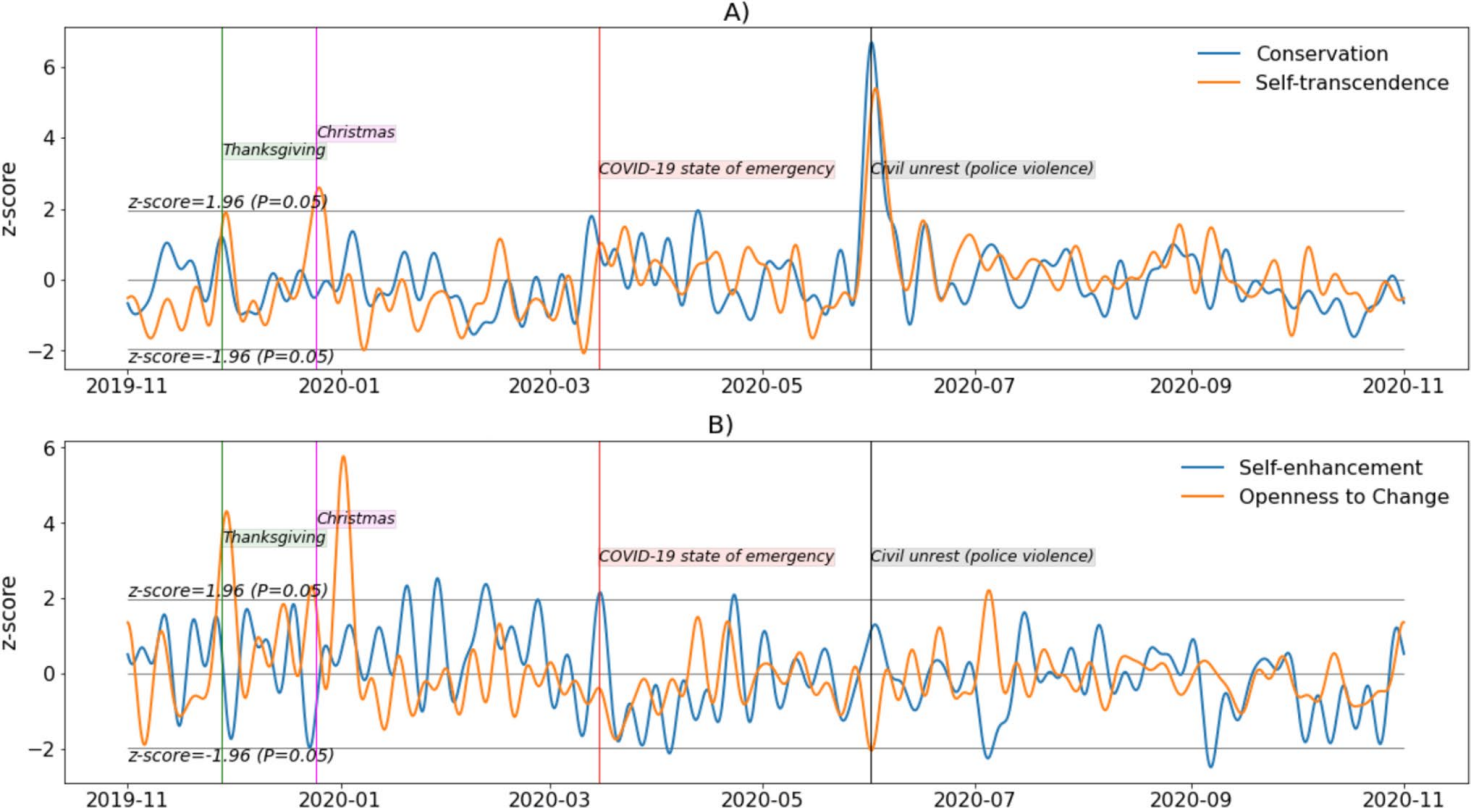
Personal value signals over 12 months (low-pass FFT filtering on periodicity beyond one week)

Around Thanksgiving, an increase can be observed in self-transcendence (two standard deviations above the average over the considered period) and openness to change (four standard deviations). An increase in self-transcendence is observed around Christmas, and the highest values for openness to change occurred near New Year’s Eve. The changes in self-transcendence observed in Fig. 6 around New Year’s Eve have been blunted by stronger filtering.

Around the time when the COVID-19 state of emergency was declared in all US states (March 15, 2020), conservation increased and reached around two standard deviations above its average for the 12 months. Values for the next month were globally higher than in the previous month. Values in self-transcendence were globally higher between March 15 and May 5 than they were between January 15 and March 15. An increase in both conservation and self-transcendence (six and five standard deviations above the average, respectively) can be observed at a time of civil unrest (e.g., the Black Lives Matter movement, starting around June 1, 2020) due to police violence (murder of George Floyd). Overall, stronger variation in self-enhancement is observed from the beginning of the period of interest to the end of the first trimester of 2020 compared with the rest of 2020. The variation in self-enhancement was less markedly relatable to events occurring during the 12 months of investigation.

- *Step 8. Change in the self-enhancement signals over 12-months: removing periodicity above 30 days.* We are now interested in signal changes over a more extended period. In Cell 8, we want to examine the result of further filtering of the data, this time removing periodicity beyond 30 days (i.e., a cutoff frequency = 1/30). We use the following code to perform the necessary filtering steps and plot the resulting signal:

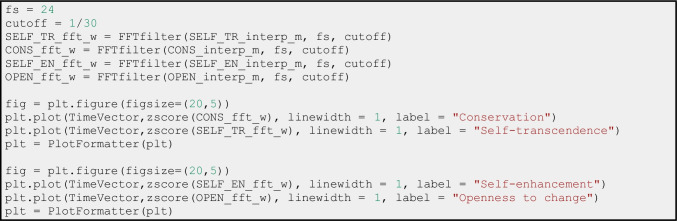

**Fig. 7:**
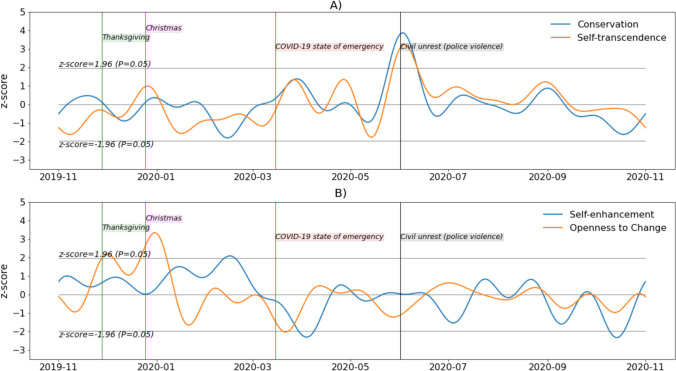
Personal value signals over 12 months (low-pass FFT filtering on periodicity beyond 30 days)

In Fig. 7, one can observe that the trend mentioned in the previous comments is still present but slightly shifted and blunted. Additional patterns are now visible, notably that conservation and self-transcendence followed very close trajectories from March 2020 to October 2020, and that self-enhancement was globally higher between November 2019 (values above the average for all of this period) and March 2020 compared with the following months (values below the average for most of the period). The lowest observed values in self-enhancement were observed in April and October 2020 (two standard deviations below the average), and the highest value was observed in February 2020 (two standard deviations above the average).

The FFT examples shown in the three previous subsections (*Steps 6–8*) could be helpful, for example, for computing both univariate and multivariate linear (i.e., correlations, Granger’s linear regression) and nonlinear (i.e., mutual information, conditional mutual information, transfer entropy) associations between the studied signals to investigate whether they are related after adjusting for periodicity. Although covering these topics is beyond the scope of this tutorial, we provide the following links to Python toolboxes and libraries specialized in such tasks, for interested readers.^5^

- *Step 9. Block bootstrapping for estimating confidence intervals (using the circular bootstrap). *The main aim of this section is to provide a tool for accessing the variability or uncertainty associated with the filtering approaches used in previous sections. To focus on a practical example, we will use the data shown in Fig. 6, in section Step 7, where we plotted FFT-filtered self-transcendence data with periodicity beyond 48 hours removed. Block bootstrapping can be used when analyzing correlated time-series data. First, we computed the residual time series by taking the difference between the raw signal and the filtered one. The resulting residual vector may have a correlated temporal structure because of the removed periodicity, where some data points may depend on previous points. We plotted the autocorrelation in Fig. 7a to verify this. As can be noted, the autocorrelation is larger for some lags than others (i.e., correlation values outside the 95% confidence interval are very likely a correlation and not a statistical fluke), indicating a periodic pattern in the residuals.

**Fig. 8:**
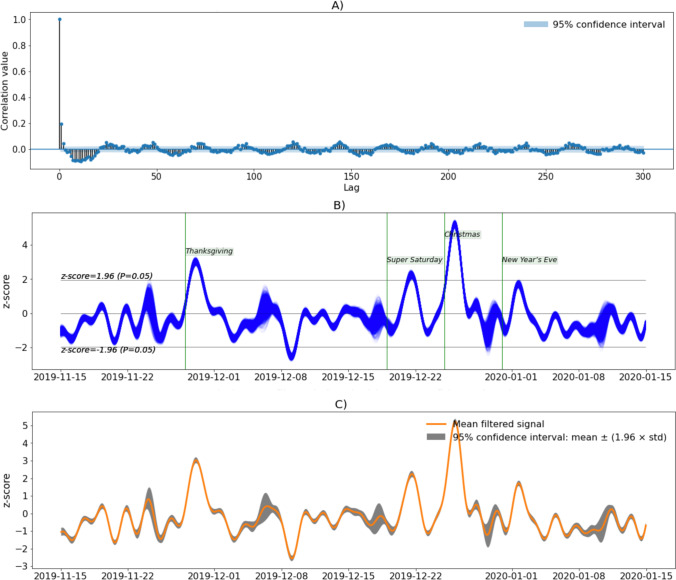
Self-transcendence. **a** Autocorrelation. **b** Block-bootstrap resampled signals. **c** Mean of the filtered signals and 95% confidence intervals

In order to preserve the temporal dependence in the residual vector, the bootstrap approach should take samples from the data in blocks rather than single observations, thus preserving the temporal dependence within each block. Therefore, we estimated the optimal block length from the residual vector in the second step. This algorithm produces optimal block lengths for the circular block bootstrap, the algorithm we use in this example. Hence, we next created 5000 copies of the original data corrupted with different noise realizations by adding 5000 versions of (circular-block) bootstrapped residuals to the denoised data. Finally, we denoised each of these 5000 signals, plotted in Fig. 8b. To quantify how stable the FFT filtering method was under these different noise realizations, the 95% confidence interval around the mean was calculated from all the repetitions (see Fig. 8c). We note that the confidence interval is relatively small, meaning that the estimated filtered signals are stable against noise. Computing such confidence intervals in this fashion is useful when there is an interest in estimating the imprecision around the estimates of a time series.

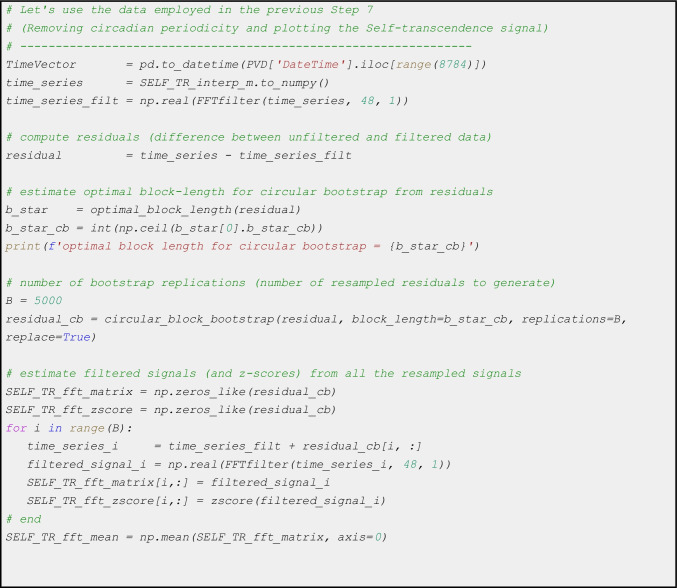

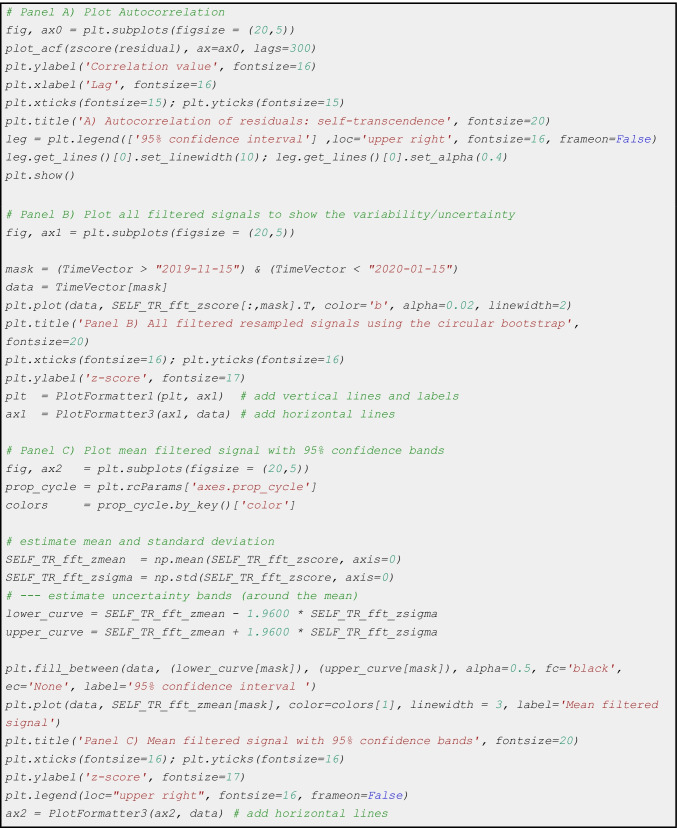





*Summary*. After granting access to Google Drive from Colab and importing the required package, we loaded the dataset into Google Colab. Then, we declared the relevant functions, interpolated the data, aggregated the data over counties, and averaged the data so each variable could be represented in a time series. Afterward, we used spectral plots to map out the power of the frequencies in the signals. Subsequently, we plotted the self-enhancement signal after removing circadian periodicity, visually showing the benefits of the FIR-filtered signal compared to the aggregated signal after interpolation. Then, we removed periodicity above one week and 30 days to examine the changes in the personal values and self-enhancement signals over 12 months. We then used block bootstrap to obtain and plot the confidence intervals.

## Conclusions

Psychologists and computer scientists have been increasingly interested in automatically processing large datasets consisting of textual documents (e.g., collected on social media; Golder & Macy, 2011). Sentiment analysis has been the most frequently used method to analyze the content of text data collected from social media (Sinnenberg et al., 2017). This has led to the discovery of large-scale collective dynamics throughout space and time and almost in real time, which would have been very difficult to identify by other methods (e.g., self-reports). For instance, temporal variations in the expression of emotions in social media (e.g., Twitter) have been studied to detect emotional contagion (Ferrara & Yang, 2015), change in public opinion (Xiong & Liu, 2014), and diurnal and seasonal mood variations (Dzogang et al., 2018; Golder & Macy, 2011; Mayor & Bietti, 2021; ten Thij et al., 2020; Wang et al., 2016). Sentiment analysis has also been used to automatically track changes in mood and behaviors in human populations over time and make predictions about future collective behaviors (Bollen et al., 2011; Golder & Macy, 2011; Tumasjan et al., 2010).

However, there has been an important mismatch between the methodological sophistication of text-mining studies in social media data analysis and how these have treated the coded text data. Most of the studies quantifying the content of textual documents have relied on coded data that have been analyzed in their initial coded form or aggregated. In this article, we proposed a tutorial for processing coded textual data as signal.

Processing coded text data as signal presents several advantages compared with commonly used techniques: it facilitates noise reduction and allows for data interpolation, detection of periodicity patterns, and visualization of data at different time frames. We presented fundamental signal analysis and processing techniques, including FIR filtering, interpolation, FFT, and FFT filtering. These are commonly applied techniques in engineering and audio analysis and production, but their use in processing regularly sampled text has been limited (e.g., Dzogang et al., 2018). We defined and explained the advantages of each of these techniques and presented a tutorial with two case studies based on two datasets of recently collected tweets (case studies A and B). We also presented changepoint detection for automatically analyzing ruptures in the signal trajectory (case study A) and the use of circular bootstrap to generate values for evaluating uncertainty around the filtered signal values (case study B).

While dataset A consisted of 317,861 emotion-coded tweets posted during the election week of 2020 in the United States, dataset B contained 7,917,884 personal value-coded tweets posted from November 2019 to October 2020, which was a period marked by major global health, social, economic, and political events notably caused by the COVID-19 pandemic. The selection of datasets for the case studies had a clear practical and pedagogical rationale. The period for case study A (dataset A) covered a single, major political event involving a progression of successive actions occurring at a short time scale (from minutes to hours) which received 24-hour live media coverage over one week. Case study A used a form of sentiment analysis (i.e., using the LIWC) well known in the field. In contrast, the period covered in case study B (dataset B) contained multiple events consisting of successive subordinate events and actions occurring at longer time scales (from days to weeks and months). It focused on investigating large-scale changes in personal values, which is unprecedented. We proposed a series of steps to apply the signal analysis and processing techniques to both case studies. Readers can quickly reproduce the steps we presented on Google Colab using the provided notebooks and datasets.

Researchers in psychology and computer science interested in analyzing automated coding of textual documents may want to apply the method we presented here to comparable datasets collected from social media (e.g., Twitter) that have been previously analyzed using traditional techniques. This could be another feasible way to test the method and experience its advantages.

Signal analysis methods developed during recent decades for characterizing and detecting nontrivial structures in regularly sampled and coded textual data can be used to reveal the nature of the underlying dynamics producing the data, and to investigate and predict the effects of short-term events on future outcomes. Our tutorial aimed to introduce basic concepts and illustrate two practical examples of its use. Additional analyses are possible by adapting related methods frequently used in econometrics (Fishman, 2013), environmental epidemiology studies (Imai & Hashizume, 2015), climate time series (Mudelsee, 2019), psychological research (Jebb et al., 2015), and physiological data from functional magnetic resonance imaging (cite: Valdés-Sosa et al., 2005). Future research will cover other advanced techniques, including multivariate autoregressive models, Granger causality, time-series analysis with explanatory variables (e.g., Maçaira et al., 2018), fluctuation analysis, and fractal geometry (e.g., see Peng et al., 1995).

### Limitations

Having precisely one observation per time point in an analyzed signal is a requirement common to the presented signal processing analyses. However, this can lead to loss of information if values are aggregated for that goal. One way to circumvent this limitation is by decreasing the period between the considered time points. Further, nothing prevents a researcher from applying the algorithms described in this tutorial to every included participant, should they have enough data at every time point. Outliers can influence values obtained by interpolation at either or both sides of the missing data. One limitation of the case studies we presented here, and in general, is that we cannot exclude the possibility that the overall observed patterns were influenced by periodicity over a larger time frame. However, more data would be needed to assess this. Null hypothesis significance testing (NHST) is not straightforward using the presented methods, because we obtained only one signal in the tutorial. Using bootstrapping, we could include 95% confidence intervals to estimate regions of significance. A simple method for NHST would be to categorize the time points in the filtered signals based on, e.g., month. Analyses of time series more in line with the aims of NHST were presented by Refinetti et al. (2007).

The tutorial is provided in Python, as this language includes powerful signal processing libraries for time-series analysis. However, similar analyses could potentially be implemented in other popular software such as R or MATLAB. We have chosen Python because of the availability of signal processing libraries, enhanced speed of execution, and the ease of use of Google Colab, which broadens the readership to non-Python users. We note that the interpretations of the patterns are more focused and less detailed than in a research paper, as this piece was intended to be a tutorial on the techniques. Because this is a tutorial and not a textbook, reviewing all existing methods relevant to text mining for psychologists and computer scientists was beyond the scope of this work. For other methods, the readers are invited to consult the following references: Weiss et al. (2015) and Ignatow and Mihalcea (2016).

## Supplementary Information

ESM 1 (DOCX 98 kb)

## Data Availability

The aggregated data for this tutorial are available on osf.io (open practices)

## Footnotes

1 Here’s an example of an introductory book to Python 3: Matthes, E. (2019). *Python crash course: A hands-on, project-based introduction to programming*. San Francisco, CA: No Starch Press.

2 For more details on FIR filtering, see https://www.sciencedirect.com/topics/engineering/fir-filters

3 For more details on importing files in Google Colab: https://colab.research.google.com/notebooks/io.ipynb

4 The mean proportion of missing values by county was 0.12 (SD = 0.12). The mean proportion of missing values by hour was 0.05 (SD = 0.12). Finally, the average maximal succession of missing values by county was 6.22 (SD = 1.73). Regarding the frequency of the sequences of missing values, the maximal sequence length was 12, and the minimum was 2, both with a frequency of 1. The most frequent lengths of sequence of missing values were 5, 6, and 7, with a frequency of 30, 35, and 32, respectively.

5 Mutual information regression: https://scikit-learn.org/stable/modules/generated/sklearn.feature_selection.mutual_info_regression.html Conditional mutual information: https://polsys.github.io/ennemi/ Copula Entropy and Transfer Entropy: https://pypi.org/project/copent/ Information-theoretic measures: https://elife-asu.github.io/PyInform/index.html Granger-Causality test, vector autoregressions: https://www.statsmodels.org/dev/index.html
