# Supplementary material for: Text as signal. A tutorial with case studies focusing on social media (Twitter)
Source: Behav Res Methods. 2022 Jul 25;55(5):2595–620. doi: 10.3758/s13428-022-01917-1 (PMC9311346; doi:10.3758/s13428-022-01917-1)
Supplement: Supplementary file 1 — (DOCX 98 kb) [file 13428_2022_1917_MOESM1_ESM.docx]

Supporting Information for

Text as Signal. A Tutorial with Case Studies Focusing on the Social Media (Twitter)

**S1.** *Existing studies relying on text as signal*

Studies relying on text as signal include the following few that have applied Fast Fourier Transform (FFT) analysis to social media text data. They have focused on detecting the emergence and interference of troll populations in local and national elections (Griffin & Bickel, 2018), identifying global patterns of human coordination in daily collective activities (Morales, Vavilala, Benito, & Bar-Yam, 2017), predicting increases in stock returns in the financial market as an effect of changes in investors’ sentiment (Mao, Counts & Bollen, 2015), identifying short-term risk of disease and providing recommendations for taking medical tests in the following days (Lafta, Zhang, Tao, Li & Tseng, 2015).

The use of FFT to detect temporal changes in emotions in social media text data in large populations has been restricted to a handful of studies to date. These studies have examined how collective mood expressed in social media is associated with searches in Wikipedia (Dzogang et al., 2017b); and is affected by circadian rhythms (Dzogang et al., 2017a, 2018). For example, Dzogang et al. (2018) notably applied Fast Fourier Transform (FFT) to millions of LIWC-coded tweets collected over four years. They examined periodic patterns of emotions and other content dimensions in the collected tweets. They found that most dimensions, including emotional ones, presented with 24-hour periodicity, and some dimensions also had a 12-hour (e.g., positive emotions) and 8-hour periodicity (e.g., affiliation).

**S2. Glossary**

*Frequency and amplitude.* Frequency refers to the number of times a given event is found in a specified period of time. If the period of time is one second, the frequency is measured in Herz (Hz; e.g., 100 Hz corresponds to 100 occurrences in one second). In signal analysis, the term amplitude refers to the size of the fluctuation of a signal, in other words how much it differs from 0.

*Periodicity.* Periodicity refers to recurring patterns observed in a signal, in other words, the frequency determinants of regular rises and falls in the data. This can be quantified using the Fast Fourier Transform.

*Composite signal*. A composite signal contains sine waves of different amplitude, frequency and phases. There are two types of composite signals: periodic and non-periodic composite signals. Their difference lies in the way in which the composite signal can be decomposed. It is possible to decompose periodic composite signals into an array of simple sine waves with discrete frequencies with integer values. Non-periodic signals can be broken down into an infinite number of simple sine waves with continuous frequencies that have actual values.

*Frequency domain and time domain*. The frequency domain representation of a signal can be opposed to its time domain representation. In the time domain representation, the signal is represented in a time series: a succession of values that vary through time. The frequency domain representation of a signal refers to the measurement of the amplitude or power of the signal at different frequencies. The passage from a time domain representation is accomplished for instance using the Fast Fourier Transform. For example, a longitudinal signal analysis before conversion would represent changes of signal over time whereas a similar kind of analysis after conversion would represent how much of the signal is present among each given frequency band. The passage from a frequency domain representation to a time domain representation is accomplished using the inverse Fast Fourier Transform.

*Imputation*. The process of replacing missing data with substituted values is called imputation. Imputation is used to overcome the problems caused by missing data in the analysis. Different forms of imputation exist, imputation by interpolation, a method suited for time series, is explained in the main text. Another frequently used form is regression imputation. Here, missing values are replaced by the values predicted by a regression model derived from data points without missing values as the dependent variable and specified predictors as the independent variables. Mean imputation was a method of predilection in the past but is currently regarded as problematic. It implies replacing missing values with the average of non-missing values

*Low-pass filtering*. Low-pass filtering deals with the use of a filter (i.e., linear operation that converts one time series into another) to lower the frequency of fluctuations in the original signal, and thus, to reduce or remove its noise. In low-pass filtering, frequencies below a certain cutoff are maintained in the output signal, while frequencies above that threshold are removed.

*High-pass filtering*. High-pass filtering is the opposite of low-pass filtering: frequencies above a certain cutoff are maintained in the output signal, while frequencies below that threshold are removed. It is employed to increase and emphasize the features of the original signal. For example, images can become sharper and show further details after the application of a high-pass filter.

*Sampling rate*. The sampling rate refers to the number of measurements by unit. For instance a sampling rate of 44,100 measurements per second is the standard sampling rate for compact discs. According to the Nyquist-Shannon theorem, a signal must be sampled at a frequency greater than or equal to twice that of the maximal frequency of interest.

*Window type*. When a FIR-filter is applied on an input signal, the signal values are transformed based on the window in use. Considering FIR-filtering, a window is a sequence of values that are multiplied with the original signal along its length to form the output signal (see for instance Figure 2). The values of the window are determined by the type of window and its length. Different types of windows exist, such as those used in the main text: triangular, hann, cosine (see Figure S1 below). Triangular windows have their highest value at the center of the window and linearly decrease towards the left and the right. Hann windows follow a formula that leads to a shape that resembles a vase, while the cosine window has a function that leads to reverse U-shape.


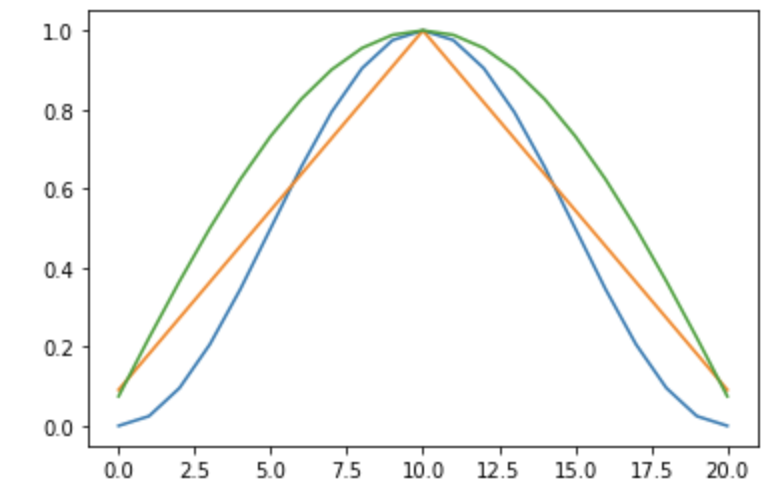


Figure S1. Representation of Triangular (orange), Hann (blue) and Cosine (green) windows.

*Linguistic Inquiry Word Count – LIWC*. The LIWC English dictionary allows for coding text into more than 80 categories related to linguistic processes (e.g., types of pronouns, types of verbs, verb tense, prepositions, quantifiers); psychological processes, especially composed of social processes (mentions of family, friends, humans); affective processes (overall score of positive and negative emotions, specific negative emotion categories); cognitive processes (insight, causation, etc.), relativity (e.g., time and motion), personal concerns (e.g., money, leisure, and religion) and spoken categories (e.g., assent and fillers). The LIWC is the most popular computer program for sentiment analysis of social media textual data (e.g., tweets) in psychology.

**S3. Background information.**

**S3A. Case study A (November 2020, Election week in the US)**

- November 1st. Texas Supreme Court rejected a petition submitted by Republican candidates to invalidate more than 120,000 drive thru votes cast in the Houston area. Trump organized rallies in several states, including Michigan, Iowa, North Carolina and Georgia. Biden and Harris campaigned in Pennsylvania and North Carolina.
- November 2nd. Trump and Pence rallied in Pennsylvania, Michigan and Wisconsin. Biden and Harris continued their campaign in Pennsylvania and Ohio.
- November 3rd. Election Day and the deadline for mail-in ballots to be received by election officials. A Federal District Court judge ordered to sweep mail facilities in 12 postal districts in important swing states, the aim of which was to find undelivered mail-in ballots. The US Postal Service reported that about 300,000 ballots it had received had not been processed for delivery. Fox News predicted that Biden would win in Arizona which was a state where Trump won in the 2016 presidential election.
- November 4th. Biden and Trump addressed their supporters several times during the day. Trump anticipated that he would file lawsuits to stop vote counting in Michigan and Pennsylvania to the US Supreme. Trump claimed that the Republican party had won the election. The Trump campaign requested a recount in Wisconsin and filed another lawsuit in Georgia to stop counting the ballots that arrived late. The Associated Press projected that Biden was the expected winner in Arizona, declared that he won in Michigan and Wisconsin and that he got closer to the 270 electoral votes to win the national election. Trump supporters protested at different election centers in the country.
- On November 5th. Courts in Georgia and Michigan dismissed Trump’s lawsuits. Trump claimed voter fraud and the Nevada Republican Party recommended criminal investigation of voter fraud to the US Attorney General. Biden told reporters that everyone should remain calm until definitive results became available.
- November 6th. Election results showed that Biden was ahead of Trump in Georgia and Pennsylvania. Vox and Business Insider announced that Biden won the election, considering his win in Pennsylvania. The Associated Press, CNN and Fox News were cautious and avoided making definitive claims. Biden preferred not to declare victory yet in an evening address to the press.
- November 7th. Most networks announced that Biden was the winner of the national election. Biden supporters celebrated the election results. Trump supporters protested in many cities throughout the country. Trump rejected the results, proceeded with the legal challenges, and claimed that the election was far from over.

**S3B. Case study B (November 2019 – October 2020, before the 1st COVID-19 wave to the beginning of the second wave)**

- November 2019. First cases of COVID-19 infections were confirmed in Wuhan, China.
- December 2019. China reported the first clusters of COVID-19 infections, beginning to raise global concerns.
- January 2020. The CDC issued a health risk warning in response to the first publicly reported clusters of COVID-19 infections in China. The WHO and China confirmed the human-to-human transmission COVID-19. The first case of COVID-19 was publicly reported in the US. The WHO declared the COVID-19 as a Public Health Emergency of International Concern. The CDC confirmed the case of human-to-human transmission in the US. The US declared a public health emergency. American nationals were evacuated from the Wuhan region.
- February 2020. The US imposed travel restrictions to and from China. First COVID-19 related death of an American in California. CDC issued a health risk warning indicating that Americans should be prepared for a local outbreak of infections. Vice President Mike Pence was appointed chair of the White House Coronavirus Task Force.
- March 2020. President Trump enacted a law (Coronavirus Preparedness and Response Supplemental Appropriations Act) for the assignment of 8.3 billion US$ to combat the propagation of COVID-19. The positive cases of COVID-19 were found in more than 100 countries worldwide. The WHO declared the COVID-19 disease a global pandemic. Dr. Anthony Fauci informed that the COVID-19 had a ten times higher mortality rate than the common flu. The CDC issued a warning announcing that the healthcare system would not be able to cope with numbers of people requiring hospital care. More than a thousand cases of COVID-19 were diagnosed in the US. The White House recommended avoiding gatherings of more than ten people. The Department of State advised against all international travel. Most states started to perform COVID-19 tests to people who needed a doctor’s approval at that point.The sharp increase in COVID-19 positive cases led nation, federal and state agencies to take urgent measures to strengthen the healthcare system (e.g., adding hospital beds, etc.). Lockdown measures were established in several states, counties, and cities. More than 100,000 COVID-19 positive cases were reported in the US only.
- April 2020. The long-term care facility industry (e.g., nursing homes) were ordered to follow CDC guidelines to prevent the rise of infections. Nursing homes were required to inform residents and family members about COVID-19 cases in their facilities. The US surpassed Italy as the country with the highest number of COVID-19 related deaths. The number of confirmed positive COVID-19 cases exceeded 1 million in the US.
- May 2020. The death toll reached 100,000 people in the US. Sharp rise in COVID-19 cases due to State economic reopening and lack of orders to wear facemasks.
- June 2020. A study reported that the real number of COVID-19 cases was almost 20 times higher than the ones confirmed.
- July 2020. The Department of State declared that the US would terminate relationships with WHO and stop funding the organization because of the questionable role it had in China when the COVID-19 pandemic started. Health experts asked for six to eight weeks lockdowns to slow down the spread of COVID-19 cases.
- August 2020. Mass gatherings took place at several states (e.g., more than 400,000 people attended the 80th Sturgis Motorcycle Rally in South Dakota) which led the propagation of the COVID-19 infection to 20 states. Over 5 million COVID-19 cases were confirmed in the US only since the beginning of the pandemic.
- September 2020. The death toll surpassed 200,000 people in the US.
- October 2020. President Trump and First Lady, Melania Trump tested positive for COVID-19 and President Trump was hospitalized for treatment with an experimental product, remdesivir and dexamethasone. The last part of the 2020 presidential election campaign was followed by a sharp increase in the number of COVID-19 cases in the states where political rallies took place.
- November 2020. Over 10 million COVID-19 cases were confirmed in the US only since the beginning of the pandemic. Pfizer announced that their vaccine against COVID-19 had an effectiveness of ninety percent. The White House announced that the COVID-19 vaccine would be free for everyone.

**S4. Other methods for the automatic coding and analysis of large datasets**

Researchers have used dictionaries (e.g., Golder & Macy, 2011), statistical language models (e.g., Kennedy et al., 2021; Jurafsky & Martin, 2009), and machine learning models (e.g., Garcia & Sikström, 2014) including neural networks (Young et al., 2018), for the automatic coding and analysis of large text datasets. Dictionaries regularly used for sentiment analysis notably include the Linguistic Inquiry and Word Count (LIWC, see Pennebaker et al., 2015), the Valence Aware Dictionary and sEntiment Reasoner (VADER, see Hutto & Gildert, 2014) and The Hu & Liu lexicon (Hu & Liu, 2004). The LIWC has been broadly employed to investigate psychologically relevant processes (e.g., emotions) expressed in textual data (Dzogang et al., 2018). The LIWC allows for coding texts in numerous categories (Pennebaker et al., 2015), including linguistic processes, psychological processes, social processes, and personal concerns. The VADER (Hutto & Gilbert, 2014) has been developed especially for the analysis of social media texts. In contrast to LIWC, VADER can handle negations, i.e. “not good” scoring opposite to “good”. Four categories result from the use of the tool: Compound (the positivity of the document), Positive, Negative, and Neutral. The Hu & Liu lexicon was developed for sentiment analysis of customer reviews. It has since been employed for the analysis of various types of texts (Hu & Liu, 2006; Qiu et al., 2011). The resulting categories are Sentiment (an overall measure of positivity), Positive and Negative.

The TF-IDF (e.g., Kennedy et al., 2021) and N-gram Language Modeling (Martin & Jurafsky, 2009) are statistical language models used for the automatic coding and analysis of text data. The TF-IDF assesses the frequency that a word has in a document and the frequency that the same word has in a set of documents. This statistical measure is useful to make a distinction between meaningful content words and function words. Thus, words that may be very frequent in a document receive a low rank in the analysis because they are also very frequent across multiple documents. The N-gram Language Modeling (Martin & Jurafsky, 2009) is used to estimate the probability of finding a word in specific language contexts. Provided the occurrence of previous words, N-gram language models predict the probability of the next word by applying corpus frequency statistics.

Latent semantic analysis (LSA, Garcia & Sikström, 2014) LSA transforms data from a high-dimensional space into a low-dimensional space where important features of the raw data are preserved. LSA is based on the fundamental assumption that semantically connected words will appear in comparable fragments of the text, frequently together or close to each other.

Machine learning models notably include Word Embeddings (Kennedy et al., 2021) and neural networks. Word Embeddings transforms textual data into numerical vectors that are then used to develop machine learning models. Word Embeddings is used to represent text in numerical vectors in a lower-dimensional space. They allow us to create similar numerical vector representations of semantically associated words and make predictions about linguistic contexts using single words. Neural Networks (NNs) are deep learning algorithms consisting of interconnected nodes with input, output and hidden layers (LeCun et al., 2015), and the connections have specific weights and thresholds. A node is only activated when outputs are above the established threshold value which leads to the propagation of data across layers of the network. NNs are trained with data, improving their reliability over time. RNNs are a type of neural network utilized to analyze data for which its sequential organization is essential (LeCun et al. 2015). An important difference with neural networks is that RNNs outputs are determined by preceding units within the sequence. Recently, a sequence-to-sequence deep learning framework (Seq2Seq) (Sutskever et al., 2014) has been demonstrated to be a successful and robust method for the analysis of time-series data, working particularly well in dynamic and complex linguistic environments. It is a type of encoder-decoder model using recurrent neural networks (RNNs) that is more efficient than classical NNs for analyzing data with a sequential organization (LeCun et al. 2015).

RNN Seq2Seq is a recent development of RNNs (Sutskever et al., 2014). They contain encoder and decoder elements. The encoder builds latent representations of sequences from previously processed input sequences. The decoder generates target sequences by converting and producing new sequences following the sequential distribution of the data received from the encoder. RNN Seq2Seq has demonstrated to be a successful and robust method for the analysis of time-series data, working particularly well in dynamic and complex linguistic environments.

**References**

Dzogang, F., Lightman, S., & Cristianini, N. (2017a). Circadian mood variations in Twitter content. *Brain and Neuroscience Advances, 1*, 2398212817744501. DOI: 10.1177/2398212817744501

Dzogang, F., Lansdall-Welfare, T, & Cristianini, N. (2017b). Seasonal fluctuations in collective mood revealed by Wikipedia searches and Twitter posts. In *2016 IEEE 16th International Conference on Data Mining Workshops* (ICDMW 2016), Institute of Electrical and Electronics Engineers (IEEE). DOI: 10.1109/ICDMW.2016.0136

Dzogang, F., Lightman, S., & Cristianini, N. (2018). Diurnal variations of psychometric indicators in Twitter content. *PloS one*, 13, e0197002. DOI: 10.1371/journal.pone.0197002

Garcia, D., & Sikström, S. (2014). The dark side of Facebook: Semantic representations of status updates predict the Dark Triad of personality. *Personality and Individual Differences*, 67, 92–96. https://doi.org/10.1016/j.paid.2013.10.001

Golder, S.A. & Macy, M.W. (2011). Diurnal and seasonal mood vary with work, sleep, and daylength across diverse cultures. *Science*, 333, 1878-1881. DOI: 10.1126/science.1202775

Griffin, C. & Brady Bickel, B. (2018). *Unsupervised machine learning of open source Russian Twitter data reveals global scope and operational characteristics.* arXiv:1810.01466v1

Hu, M. & Liu, B. (2004). Mining and summarizing customer reviews. In *Proceedings of the tenth ACM SIGKDD international conference on Knowledge discovery and data mining* (pp. 168–177), Seattle: ACM. DOI: 10.1145/1014052.1014073

Hutto, C., & Gilbert, E. (2014). VADER: A parsimonious rule-based model for sentiment analysis of social media text. *Proceedings of the International AAAI Conference on Web and Social Media, 8(1)*, 216-225.

Jurafsky, D., & Martin, J. H. (2009). *Speech and language processing: an introduction to natural language processing, computational linguistics, and speech recognition*. Upper Saddle River, N.J.: Pearson Prentice Hall. ISBN: 9780131873216 0131873210

Kennedy, B., Ashokkumar, A., Boyd, R. L., & Dehghani, M. (2021). *Text analysis for Psychologp: Methods, principles, and Practice*. DOI: 10.31234/osf.io/h2b8t

Lafta, R., Zhang, J., Tao, X., Li, Y., & Tseng, V.S. (2015). An intelligent recommender system based on short-term risk prediction for heart disease patients. *In 2015 IEEE/WIC/ACM International conference on web intelligence and intelligent agent technology (WI-IAT),* vol. 3, (pp. 102–105). DOI: 10.1109/WI-IAT.2015.47

LeCun, Y., Bengio, Y. & Hinton, G. (2015). Deep learning. *Nature, 521*, 436–444. DOI: 10.1038/nature14539

Mao, H., Counts, S., & Bollen, J. (2015). *Quantifying the effects of online bullishness on international financial markets.* Frankfurt a. M.: European Central Bank (ECB), DOI: 10.2866/943096

Morales, A. J., Vavilala, V., Benito, R. M., & Bar-Yam, Y. (2017). Global patterns of synchronization in human communications. *Journal of the Royal Society, Interface*, *14*, 20161048. DOI: 10.1098/rsif.2016.1048

Pennebaker, J. W., Boyd, R. L., Jordan, K., & Blackburn, K. (2015). *The development and psychometric properties of LIWC2015*. Austin, TX: University of Texas at Austin.

Qiu, G., Liu, B., Bu, J., & Chen, C. (2011). Opinion word expansion and target extraction through double propagation. *Computational Linguistics, 37*(1), 9-27.

Sutskever, I., Vinyals, O., & Le, Q.V. (2014). *Sequence to sequence learning with neural networks.* https://arxiv.org/abs/1409.3215v3

Young, T., Hazarika, D., Poria, S., & Cambria, E. (2018). Recent trends in deep learning

based natural language processing. *IEEE Computational Intelligence Magazine, 13*(3), 55–75. DOI : 10.1109/MCI.2018.2840738
